# Assessment and Diagnosis of Musculoskeletal Shoulder Disorders over the Internet

**DOI:** 10.1155/2012/945745

**Published:** 2012-11-05

**Authors:** Leah Steele, Hannah Lade, Stephanie McKenzie, Trevor G. Russell

**Affiliations:** Division of Physiotherapy, School of Health and Rehabilitation Sciences, University of Queensland, Brisbane, QLD 4072, Australia

## Abstract

Shoulder disorders are common, debilitating, and represent a considerable burden on society. As primary contact practitioners, physiotherapists play a large role in the management and rehabilitation of people with these conditions. For those living outside of urban areas, however, access to physiotherapy can be limited. The aim of this study was to evaluate the validity and reliability of using a telerehabilitation system to collect physical examination findings and correctly identify disorders of the shoulder. Twenty-two participants with 28 shoulder disorders were recruited and underwent a face-to-face physical examination and a remote telerehabilitation examination. Examination findings and diagnoses from the two modes of assessment were used to determine validity and reliability of the new method. Diagnostic agreement and agreement on individual findings between the two methods were found to be consistent with the reliability of conventional assessment methods. This study provides important preliminary findings on the validity and reliability of musculoskeletal examinations conducted via telerehabilitation.

## 1. Introduction

Shoulder pain is common in society, with 7%–27% of the adult population experiencing shoulder pain at any one time, and 7%–67% of people experiencing shoulder pain in their lifetime [1]. Of first episode shoulder pain clients, 50% will continue to have symptoms 18 months later [2], making shoulder pain the third most common reason for primary care consultation, after back and neck complaints [3]. Accurate and effective assessment and treatment of shoulder conditions is therefore important to health care practitioners. Physiotherapists often assess and treat clients with shoulder pain, and there exists considerable evidence of effective physiotherapy treatments for many shoulder disorders [3–8], with conservative physiotherapy management shown to provide up to an 88% improvement in shoulder function in the long term [9]. 

Unfortunately, people living in rural and remote areas of Australia have limited access to physiotherapy services, a phenomenon observed also in the USA [10–12]. Telerehabilitation, the provision of rehabilitation services via the internet, is one potential service delivery model which may improve access to physiotherapy services in rural and remote areas. However, relatively little research has been conducted into the validity and reliability of telerehabilitation for the assessment and treatment of musculoskeletal disorders [13, 14]. To establish the concurrent validity of such services, research must prove that measurements taken using telerehabilitation are the same or similar as those that are made in the traditional face-to-face manner. The reliability of such assessments should also be established prior to the wide scale adoption of telerehabilitation into the community. 

Research has established the validity and reliability of telerehabilitation to assess joint range of motion (ROM) at the knee, wrist, elbow, forearm supination, and pronation as well as all movements at the shoulder [15–17]. Studies have also proven the validity of remote assessment of quadriceps muscle strength and limb girth [15] and functional analysis of gait [15, 18]. However, to perform a comprehensive musculoskeletal assessment for a complex joint such as the shoulder, further research should be performed into the other tests commonly required for a shoulder examination, such as special orthopaedic and neural system tests.

To date, only two studies have used physical outcome measurements from a telerehabilitation consultation to establish the validity and reliability of telerehabilitation for remote diagnosis [19, 20]. Both studies focused on lower limb disorders, the first on the ankle^35^ and the second on nonarticular disorders of the lower limb^36^. The two studies reported 80% and 79% exact agreement for diagnosis respectively, with the percentage agreement for their physical examination findings ranging from 76.4% to 99.9%. The authors of the studies concluded that the telerehabilitation assessments appeared to be accurate and valid and demonstrated high intra- and interrater reliability. This claim is made in the context of research that shows that the agreement between two face-to-face examinations was similar in magnitude. Indeed, it should be noted that in the shoulder, research has demonstrated that the inter-rater reliability of face-to-face examinations of this complex joint may be as low as 46% Bamji et al. [21]. Relative poor agreement between face-to-face examinations must therefore be considered when investigating the use of telerehabilitation for assessment and diagnosis for the upper limb. This study has three aims: (1) to evaluate the use of a telerehabilitation system to formulate valid and reliable diagnoses of shoulder disorders; (2) to establish the validity and reliability of the individual physical examination findings via the telerehabilitation system; (3) to examine the satisfaction of the participants with the use of the system for their physiotherapy examination. We hypothesised that telerehabilitation will be valid and reliable in generating physical examination findings and can be used by examiners to create valid and reliable diagnoses for musculoskeletal problems of the shoulder.

## 2. Methods

### 2.1. Participants

A total of 22 participants with 28 reports of shoulder pain (some had both left and right sided problems, which were considered independently) were recruited over a one-month period for this study. Participants were recruited from within the community of a large tertiary university in Brisbane, Queensland (students and staff), and the university's musculoskeletal and sports injury physiotherapy clinic. Participants were included if they were over 18 years old, English speaking, and possessed an adequate level of cognition and communication to complete a full physiotherapy assessment. The exclusion criteria included poor vision or hearing and concomitant medical conditions such as severe respiratory or cardiac conditions that would prevent participants from safely completing the examinations. All participants volunteered for the study and provided signed informed consent after receiving written and verbal explanations on how the session would progress. Approval was gained from the relevant Medical Research Ethics Committee before commencement of the project.

### 2.2. Examiners

A convenience sample of three final year physiotherapy honours students were the examiners for this study. They had all completed the musculoskeletal training components of their degree prior to the project. During the study, the students each had access to separate independent, experienced clinical educators for the purposes of clinical reasoning assistance. For example, the students were able to ask questions about and discuss the interpretation of assessment findings. The students were blinded to each other's assessments and results to avoid bias. 

### 2.3. Equipment

Remote patient interviews and physical examinations were performed using the eHAB Telerehabilitation system (Neorehab, Brisbane, QLD, Australia). This system uses a wireless 3G Internet connection (Telstra Next G) and allows videoconferencing as well as a battery of physical measurement tools, as described elsewhere in the literature [22].

### 2.4. Procedure

Participants attended a single 1.5 hour session at the University of Queensland, during which a patient interview, a face-to-face physical examination and a remote physical examination were undertaken. The order of examinations was randomly determined upon the participant's arrival for the session, using a balanced block design of size four. The randomisation code was determined prior to the commencement of the study and was administered by an author (T. G. Russell) who was not involved in performing the participant assessments. The balanced block design ensured that for every four participants recruited, two were examined via telerehabilitation first and these were performed by different examiners. The examiners that were randomly assigned to the first physical examination also conducted the patient interview in the same mode as they were to perform the physical examination. This patient interview was simultaneously observed passively by the alternative examiner in the mode of assessment they were assigned to use for their physical examination. Using this method, the participant only underwent one patient interview. All patient interviews and remote physical examinations were recorded at the time of assessment using the eHAB telerehabilitation system, to be used later for reliability analysis.

The patient interview consisted of questions traditionally asked during a physiotherapy patient interview. The face-to-face physical examination was conducted in the conventional manner with the physiotherapist in the room with the participant and utilising tests they felt appropriate according to their clinical reasoning. Tests included postural analysis, joint palpation, range of motion (ROM) testing at the shoulder and adjacent joints, static muscle tests (SMT's), special orthopaedic tests and neural testing. Neural testing involves assessing the response of the neural system to movement at the joints that the nerve crosses.

**Figure 1:**
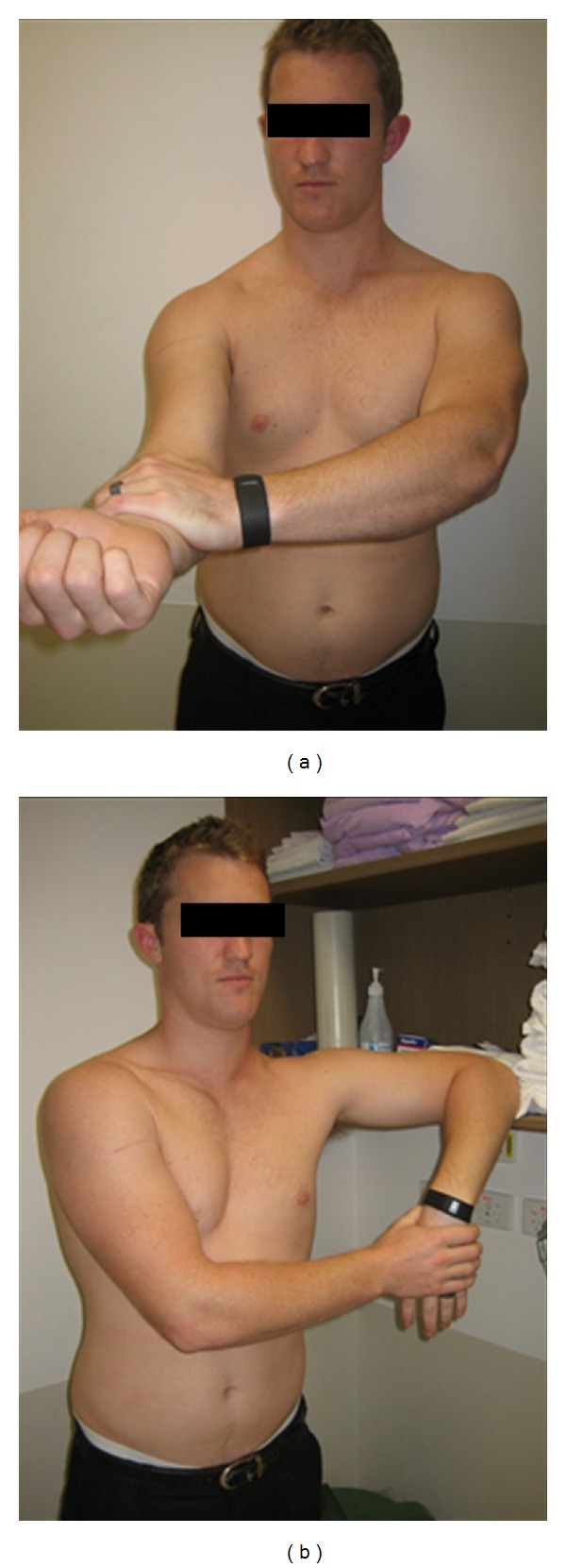
(a) Participant performing Speeds test. (b) Participant performing Hawkins-Kennedy.

The remote examination was conducted with the examiner in another room, communicating with the participant via the eHAB telerehabilitation system. Similarly to the face-to-face examination, the remote examiners lead the participant through tests they felt appropriate for their particular presentation, according to their clinical reasoning. As the examiner had no physical contact with the participant, the examiners verbally lead the participant through the tests, demonstrating on themselves for the participants to copy. Many of the tests used the participants other arm and objects that can be found in the home; for example; when conducting “Speeds test,” a test designed to assess for pathology of the long head of biceps, the participants held their arm out straight in front of them and used their other arm to apply pressure in a downward force (Figure 1(a)), or when conducting the Hawkins-Kennedy impingement test, participants used a nearby surface at the level of the shoulder (Figure 1(b)). 

To determine the validity and reliability of the pathoanatomical diagnosis, the diagnoses were compared by two blinded, independent, experienced clinicians and recorded as the same, similar, or different (Table 2).

To allow statistical analysis, the physical examination findings were recorded and coded according to a system developed by the examiners for this study (see example in Table 1). Some outcome measures were recorded in a binary format; for example, strength was recorded as full or reduced; orthopaedic tests were recorded negative or positive. Others were recorded on a categorical scale; for example, pain on palpation of the shoulder joint (joint assessment) was given a value from zero to ten, while bruising, muscle wasting, and postural deviation were recorded on a severity scale from nil to severe. Upon completion of the two physical examinations, participants filled out a satisfaction survey.

The videorecordings of the patient interview and physical examinations captured from the original telerehabilitation examination were used to evaluate the reliability of the telerehabilitation assessments. Inter-rater reliability was established by a third examiner independently analysing the videorecordings and formulating a diagnosis. Intra-rater reliability was established by the original remote examiner reanalysing the videos after a 6-week waiting period. 

### 2.5. Measures

All examiners recorded a primary diagnosis of the participants presenting condition in the form of a patho-anatomical structure (e.g., supraspinatus tear), condition (e.g., adhesive capsulitis), or in descriptive terms of a movement dysfunction (e.g., scapular dyskinesia). A system diagnosis was also nominated, referring to the anatomical system (muscle, bone, articular, neural, and other) responsible for the primary condition. In addition to these, the physical examination findings, which were coded into either binary or categorical data, were recorded to enable the statistical comparison of the individual examination procedures.

The questionnaire that was completed by participants at the conclusion of the study used a 10 cm visual analogue scale (VAS) to get their opinion on (1) how beneficial participants rated the Internet examination, (2) recommend to a friend who was unable to travel, (3) as good as the “face-to-face” examination, (4) visual clarity, (5) Audio clarity, and (6) overall satisfaction with the Internet examination. 

### 2.6. Data Analysis

Data was analysed using Medcalc, version 10.4.8.0 (Medcalc Software, Ghent, Belgium) and SPSS, version 19 (IBM, Armonk, NY, USA). A *P* value of < 0.05 was used to denote significance.

Validity and reliability were analysed for all data gathered during the examination. The validity was established by comparing the face-to-face examiners' findings to telerehabilitation examiners' findings for each participant. Similarly, inter-rater reliability was assessed by comparing the original telerehabilitation examiners' findings to second telerehabilitation examiners' findings, and intra-rater reliability was assessed by comparing the first and second findings of the original telerehabilitation examiner. 

The validity and reliability of the pathoanatomical diagnoses, as mentioned above, were recorded by two-blinded, independent, experienced clinicians as the same, similar, or different. If the independent clinicians differed in their opinion, a third experienced clinician arbitrated until consensus was obtained. These findings were then analysed using descriptive statistics. Similarly, the validity and reliability of the systems' diagnosis were analysed using percent agreement and *χ* ^2^ statistics. 

The findings during the physical examination were recorded as described in Table 1. Assessments which produced binary data (full/reduced, negative/positive) were analysed using percentage agreement and *χ* ^2^ statistics. The assessments which produced an outcome on a scale (categorical data) were analysed using percentage agreement (exact and close, with close determined as one rating above or below compared rating) and quadratically weighted kappa (*κ*). The strength of agreement was appraised according to the guidelines stipulated by Landis and Koch [23].

The questionnaire data was measured in millimetres on the 10 cm scale by the same person, using the same ruler, and was analysed using descriptive statistics.

## 3. Results

### 3.1. Participants

This study included 16 males and 6 females who presented to the clinic reporting a problem with their shoulder. The participants' ages ranged from 18 to 60 years old, with an average of 30.7 years, and a standard deviation of 14.2 years. As previously described, a number of participants had both left and right sided problems which were considered independent in the study, producing a total number of 28 assessments. The order of assessment (Face-to-face or Telerehabilitation) first did not appear to be a factor in the results (Wilcoxon Signed Rank Test; *Z* = 0.91, *P* = 0.37)

### 3.2. Pathoanatomical Diagnosis

Results for the analysis of the pathoanatomical diagnoses are presented in Table 3. Moderate agreements were demonstrated for the combined same and similar results for validity (59.72% agreement). Reliability achieved stronger results with substantial agreements that achieved for inter-rater reliability (73.08%) and almost perfect agreements for intra-rater reliability (100%) that combined same and similar results. 

### 3.3. Systems' Diagnosis

The results for the primary systems' diagnosis analysis are presented in Table 4 and demonstrate substantial validity (78.6% agreement; *χ* ^2^ = 35.70; *P* < 0.001), with even stronger results for intra-rater (82.1% agreement; *χ* ^2^ = 38.05; *P* < 0.001) and inter-rater (82.1% agreement, *χ* ^2^ = 41.60; *P* < 0.001) reliability.

### 3.4. Physical Examination Findings

Analysis of the physical examinations which produced binary data is presented in Table 4. Validity analysis of physical examination findings produced strong agreements for most outcome measures, with the highest agreement for ROM (87.4% agreement; *χ* ^2^ = 30.782; *P* < 0.001), and the lowest significant agreement for nerve testing (56.1% agreement; *χ* ^2^ = 6.291; *P* = 0.012). Joint assessment findings were found to have poor agreement and failed to reach statistical significance (64.4% agreement, *χ* ^2^ = 0.762, *P* = 0.383). Reliability analysis of physical examination parameters was found to be very high across all binary measures, ranging from 66.9% to 98.3% agreement (7.204 < *χ* ^2^ < 1795.945, all *P* < 0.05).

Analysis of the assessment items which produced categorical data is presented in Table 5, demonstrating fair agreement for pain ratings (67.7% exact agreement and 76.8% exact and close agreement, *κ* = 0.50), and substantial agreement for the severity rating scale (80.8% exact agreement, 96.0% exact and close agreement, *κ* = 0.66). Intra-rater (94.1% exact, 97.2% exact and close, *κ* = 0.95), and inter-rater (93.6%-exact, 97.2% exact and close, *κ* = 0.95), reliability analysis for pain ratings demonstrated almost perfect agreement, with similar agreements for severity scale ratings (88.5% exact agreement, 97.7% exact and close agreement, *κ* = 0.83; 85% exact agreement, 99.2% exact and close agreement, *κ* = 0.83), respectively.

### 3.5. Patient Satisfaction

**Figure 2:**
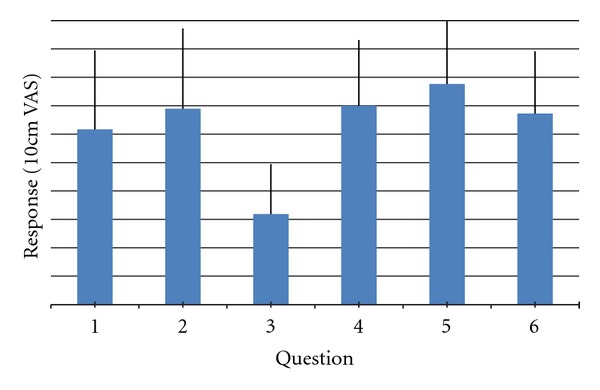
Patient satisfaction questionnaire findings. Questions: (1) How beneficial participants rated the Internet examination, (2) recommend to a friend who was unable to travel, (3) as good as the ‘face-to-face' examination, (4) visual clarity, (5) audio clarity, (6) overall satisfaction with the Internet examination.

Results for patient satisfaction are presented in Figure 2, demonstrating that the participants were very satisfied with the telerehabilitation mode of assessment, with average ratings of 6.8/10.

The use of telerehabilitation to diagnose clients with shoulder disorders appears to be both valid and reliable as well as acceptable to participants. This study represents an important first step in obtaining evidence for the use of telerehabilitation for clients that otherwise would find access to physiotherapy services difficult.

Systematic reviews investigating the physical examination tests used when assessing the shoulder in the face-to-face method have found that they do not demonstrate high levels of validity or reliability [24–27] and are affected by information gathered during the patient interview [26]. A meta-analysis by Hegedus et al. [27] concluded that many of the shoulder tests have limited diagnostic accuracy; however, it has been suggested that some (many of those employed in the present study) can be used as a screen for certain shoulder pathologies. Poor reliability findings have been reported for tests used in physical examinations in general, with similar results in many other areas of the body [24]. In light of the poor reliability of shoulder examination tests, it has been suggested that expert clinicians consider their results within the context of the patient interview and patterns of physical examination findings, rather than relying on one key finding or outcome measure [24, 28, 29]. Considering the difficult nature of physical examinations of the shoulder and the limited reliability seen in face-to-face studies, the strong agreement found in this study between face-to-face and telerehabilitation assessment is convincing evidence for the validity of online physical assessments of the shoulder.

The percentage agreement for diagnoses obtained for validity in the study is fair. Although exact diagnoses agreement was low (18.5%), the combined same and similar results demonstrate moderate agreement (59.7%). Stronger agreements were achieved for inter-rater reliability (73.1%) and intra-rater reliability (100%) combining same and similar agreements. Previous research on the reliability of face-to-face examinations of the shoulder reveals conflicting results [21, 29–31]. Some studies reported very good agreements (Pellecchia et al. [31], 90.5% agreement, *κ* = 0.875; Carter et al. [29], 80% agreement, *κ* = 0.664), whereas others report poorer agreement rates (De Winter et al. [30]; 60% agreement, *κ* = 0.45, Bamji et al. [21]; 46% agreement, no kappa recorded). Poor diagnostic agreements for the shoulder are not particularly surprising, as there exists no generally accepted explanation for the aetiology and pathogenesis of many shoulder disorders [24, 30, 32]. The disagreements typically arise when patients have increased pain severity, more than one problem, have bilateral involvement, and when examiners vary on their interpretation of physical examination signs [21, 29–31]. Our study asked examiners to write a free text diagnosis, while the previous studies all required examiners to assign participant diagnoses according to distinct groups. Within this context, and in light of our fair diagnostic agreement results, it appears that the introduction of a telerehabilitation system does not compound the difficulties already faced for diagnosis of the shoulder in the clinical setting. 

Despite the current evidence which indicates that reliability is poor for the physical examination tests of the shoulder, high inter-rater and intra-rater reliability rates were recorded for the tests in the current study. It could be said that any differences in diagnoses could potentially be explained by differing clinical reasoning processes between examiners rather than a factor of the mode of assessment. This has been reported in previous diagnostic accuracy literature, with studies finding inherent differences between clinicians when faced with the same clinical information [33, 34]. Bamji et al. [21] found that even when examiners discussed and agreed on all the clinical signs for the participants, they still reached a different diagnosis in 22% of cases. 

The findings of this study are similar to previous telerehabilitation diagnostic accuracy studies [19, 20]. Two prior studies reported high levels of validity and reliability for the use of a telerehabilitation system in the diagnosis of ankle disorders and nonarticular disorders of the lower limb. The percentage agreement findings in these studies for the validity of systems diagnosis (at 80% and 79% resp.) were comparable to this study (at 78.1%). The present study recorded much higher Chi-squared values for systems diagnosis (validity and reliability), with the previous studies ranging from 4.27 < *χ* ^2^ < 13.46, compared to the current studies results ranging from 35.70 < *χ* ^2^ < 41.6. This may be explained by the fact that the previous two studies grouped the binary and categorical examination findings for statistical analyses, while the current study kept them separate in order to obtain validity and reliability information on each specific test. All studies, however, report high agreements for the physical examination recordings. The previous studies report categorical data exact agreement results ranging from 76.4% to 94.5%, and binary data agreement ranging from 82.9% to 99.9%. These findings are comparable to the present study's findings, with our physical examination analysis recording agreements from 56.1% to 94.1%. 

On closer examination of the poor results of the joint assessment findings in this study (64.4% agreement, *χ* ^2^ = 0.762, *P* = 0.383), it was noted that there were considerable differences between the examiners in their method of recording results. One examiner did not use this section at all, and the others did so sporadically and without a systematic approach. Additionally, we obtained relatively poor agreement results for the assessment of the neural system. We believe that the reasons for this may be twofold. Firstly, the neurodynamic tests involved very complex movements at many joints within a number of planes, making it difficult to verbally describe. Secondly, neural testing was always performed last during the examination, after a battery of previous tests, and the participants were often fatigued by this point. These difficulties could potentially be avoided in the future by performing the tests earlier in the session and by creating a premade instruction video to send to the participants that is clear and easy to follow. 

One aspect of telerehabilitation which has been widely discussed and investigated in the literature is its acceptability to clients and health care professionals. A systematic review of reviews on telemedicine by Ekeland et al. [35] found promising evidence of high client and health professional satisfaction ratings for telerehabilitation. It has been proposed that its utilisation can empower clients, giving them higher confidence levels and a deeper understanding of their condition, leading to improved health outcomes [35–37]. Analysis of the participants comments in the present study revealed that the face-to-face assessment was preferable to the remote assessment, however, that participants would recommend the internet examination to a friend who was unable to travel for treatment. As telerehabilitation aims to provide an alternative when physical distance or disability makes travel difficult, this is an encouraging result.

There were a number of limitations in this study. The inexperience of the examiners and their lack of “real world” clinical experience may have influenced their ability to formulate accurate diagnoses. Students were primarily used as this was an unfunded trial. Despite this, good results were still obtained, which is promising as it is reasonable to anticipate that these results may be improved when repeated with experienced physiotherapists. Secondly, the demographic of the participants, although spanning across a wide range of ages, was restricted to the university community, and thus the results of this study can only be generalised to other populations with caution. These limitations should be addressed in future research using experienced examiners with larger sample sizes and using people from a varied demographic background. Additionally, future research should investigate the ability of the system to clinically monitor the progress of a client through the course of their rehabilitation.

## 4. Clinical Messages

- The use of telerehabilitation to gather information and diagnose clients with shoulder disorders appears to be both valid and reliable.
- This is an important first step in obtaining evidence for the use of telerehabilitation for clients that otherwise would find access to physiotherapy services difficult.

## Figures and Tables

**Table 1:** Example of physical examination recording and coding.

Test	Rating	Data coding
Postural deviation	Normal/mild/moderate/ severe	0/1/2/3/4
ROM (e.g., (L) shoulder flexion)	Full/restricted	0/1
Limiting factor (to ROM)	Nothing/pain/stiffness/ pain and stiffness	0/1/2/3
Pain on active ROM	Scale from 0–10	0/1/2/3/4/5/6/7/ 8/9/10
Strength (e.g., (L) middle deltoid)	Full/reduced	0/1
Pain on SMT (e.g., middle deltoid)	Pain ISQ/pain Increase	0/1
Special orthopaedic test (e.g., O'Briens)	Negative/positive	0/1

(L): Left; ROM: range of motion; ISQ: in status quo.

**Table 2:** An example of comparison of pathoanatomical diagnoses.

Participant no.	Diagnosis 1	Diagnosis 2	Result
a	Supraspinatus tendinitis and functional subacromial impingement with neural tightness and mechanosensitivity	Left functional subacromial impingement syndrome due to rotator cuff insufficiency (mainly supraspinatus tendinopathy) plus neural mechanosensitivity (left median nerve)	Same

b	Right chronic acromioclavicular joint pain due to degeneration	Right AC joint arthrosis with rotator cuff insufficiency and possible supraspinatus tendinopathy	Similar

c	Left mild glenohumeral joint laxity and rotator insufficiency	Left rotator cuff insufficiency and functional impingement due to overload	Different

**Table 3:** Results from the analysis of pathoanatomical diagnosis agreement.

	Same	Similar	Different
Validity	18.52%	40.74%	40.74%
Interrater reliability	23.08%	50%	26.92%
Intrarater reliability	40.74%	59.26%	0%

**Table 4:** Results from the analysis of physical examinations producing binary data.

Test		Percentage agreement	Chi-squared	*P* value
	Validity	78.600	35.703	<0.001
Systems diagnosis	Intra-rater	82.100	38.050	<0.001
	Inter-rater	82.100	41.600	<0.001

	Validity	87.400	30.782	<0.001
ROM	Intra-rater	95.800	393.950	<0.001
	Inter-rater	92.100	298.492	<0.001

	Validity	75.900	54.765	<0.001
Special orthopaedic tests	Intra-rater	88.700	185.337	<0.001
	Inter-rater	88.100	209.515	<0.001

	Validity	81.700	70.867	<0.001
Pain response to SMT	Intra-rater	96.800	510.596	<0.001
	Inter-rater	98.300	618.832	<0.001

	Validity	56.100	6.291	0.012
Nerve ROM and sensitisation	Intra-rater	87.100	76.582	<0.001
	Inter-rater	66.900	7.204	0.007

	Validity	87.100	31.546	<0.001
Strength	Intra-rater	97.300	585.732	<0.001
	Inter-rater	95.400	476.739	<0.001

	Validity	64.400	0.762	0.383
Joint assessment	Intra-rater	85.900	51.004	<0.001
	Inter-rater	90.500	43.990	<0.001

	Validity	68.100	320.182	<0.001
Limiting factor	Intra-rater	88.900	1795.945	<0.001
	Inter-rater	87.000	1549.903	<0.001

ROM: range of motion; SMT: static muscle test.

**Table 5:** Results from the analysis of physical examinations producing categorical data.

Test		Percentage agreement			Weighted kappa	Strength of agreement
		Exact	Close	Exact and close		
	Validity	67.70	9.10	76.80	0.50	Fair
Pain	Intra-rater reliability	94.10	3.10	97.20	0.95	Almost Perfect
	Inter-rater reliability	93.60	3.60	97.20	0.95	Almost Perfect

	Validity	80.80	15.20	96.00	0.66	Substantial
Severity scale	Intra-rater reliability	88.50	9.20	97.70	0.83	Almost Perfect
	Inter-rater reliability	85.00	14.20	99.20	0.83	Almost Perfect

## References

[1] Luime JJ, Koes BW, Hendriksen IJM (2004). Prevalence and incidence of shoulder pain in the general population; a systematic review. *Scandinavian Journal of Rheumatology*.

[2] Croft P, Pope D, Silman A (1996). The clinical course of shoulder pain: prospective cohort study in primary care. *British Medical Journal*.

[3] Green S, Buchbinder R, Hetrick S (2003). Physiotherapy interventions for shoulder pain. *Cochrane Database of Systematic Reviews*.

[4] Bennell K, Wee E, Coburn S (2010). Efficacy of standardised manual therapy and home exercise programme for chronic rotator cuff disease: randomised placebo controlled trial. *British Medical Journal*.

[5] Crawshaw DP, Helliwell PS, Hensor EM, Hay EM, Aldous SJ, Conaghan PG (2010). Exercise therapy after corticosteroid injection for moderate to severe shoulder pain: large pragmatic randomised trial. *British Medical Journal*.

[6] Carette S, Moffet H, Tardif J (2003). Intraarticular corticosteroids, supervised physiotherapy, or a combination of the two in the treatment of adhesive capsulitis of the shoulder: a placebo-controlled trial. *Arthritis and Rheumatism*.

[7] Hay EM, Thomas E, Paterson SM, Dziedzic K, Croft PR (2003). A pragmatic randomised controlled trial of local corticosteroid injection and physiotherapy for the treatment of new episodes of unilateral shoulder pain in primary care. *Annals of the Rheumatic Diseases*.

[8] Van Peppen RPS, Kwakkel G, Wood-Dauphinee S, Hendriks HJM, Van der Wees PJ, Dekker J (2004). The impact of physical therapy on functional outcomes after stroke: what’s the evidence?. *Clinical Rehabilitation*.

[9] Ginn KA, Cohen ML (2004). Conservative treatment for shoulder pain: prognostic indicators of outcome. *Archives of Physical Medicine and Rehabilitation*.

[10] Smith T, Cooper R, Brown L, Hemmings R, Greaves J (2008). Profile of the rural allied health workforce in Northern New South Wales and comparison with previous studies. *Australian Journal of Rural Health*.

[11] Wilson RD, Lewis SA, Murray PK (2009). Trends in the rehabilitation therapist workforce in underserved areas: 1980–2000. *Journal of Rural Health*.

[12] Dixon J, Welch N (2000). Researching the rural-metropolitan health differential using the ’social determinants of health’. *The Australian Hournal of Rural Health*.

[13] Russell TG (2007). Physical rehabilitation using telemedicine. *Journal of Telemedicine and Telecare*.

[14] Hersh WR, Hickam DH, Severance SM, Dana TL, Krages KP, Helfand M (2006). Diagnosis, access and outcomes: update of a systematic review of telemedicine services. *Journal of Telemedicine and Telecare*.

[15] Russell TG, Wootton R, Jull GA (2002). Physical outcome measurements via the Internet: reliability at two Internet speeds. *Journal of Telemedicine and Telecare*.

[16] Russell T (2007). Goniometry via the internet. *Australian Journal of Physiotherapy*.

[17] Hoffmann T, Russell T, Cooke H (2007). Remote measurement via the Internet of upper limb range of motion in people who have had a stroke. *Journal of Telemedicine and Telecare*.

[18] Russell TG, Jull GA, Wootton R (2003). The diagnostic reliability of Internet-based observational kinematic gait analysis. *Journal of Telemedicine and Telecare*.

[19] Russell T, Truter P, Blumke R, Richardson B (2010). The diagnostic accuracy of telerehabilitation for nonarticular lower-limb musculoskeletal disorders. *Telemedicine Journal and E-Health*.

[20] Russell TG, Blumke R, Richardson B, Truter P (2010). Telerehabilitation mediated physiotherapy assessment of ankle disorders. *Physiotherapy Research International*.

[21] Bamji AN, Erhardt CC, Price TR, Williams PL (1996). The painful shoulder: can consultants agree?. *British Journal of Rheumatology*.

[22] Russell TG, Buttrum P, Wootton R, Jull GA (2011). Internet-based outpatient telerehabilitation for patients following total knee arthroplasty: a randomized controlled trial. *Journal of Bone and Joint Surgery Series A*.

[23] Landis JR, Koch GG (1977). The measurement of observer agreement for categorical data. *Biometrics*.

[24] May S, Chance-Larsen K, Littlewood C, Lomas D, Saad M (2010). Reliability of physical examination tests used in the assessment of patients with shoulder problems: a systematic review. *Physiotherapy*.

[25] Calvert E, Chambers GK, Regan W, Hawkins RH, Leith JM (2009). Special physical examination tests for superior labrum anterior posterior shoulder tears are clinically limited and invalid: a diagnostic systematic review. *Journal of Clinical Epidemiology*.

[26] Bertilson BC, Grunnesjö M, Strender LE (2003). Reliability of clinical tests in the assessment of patients with neck/shoulder problems—impact of history. *Spine*.

[27] Hegedus EJ, Goode A, Campbell S (2008). Physical examination tests of the shoulder: a systematic review with meta-analysis of individual tests. *British Journal of Sports Medicine*.

[28] May S, Greasley A, Reeve S, Withers S (2008). Expert therapists use specific clinical reasoning processes in the assessment and management of patients with shoulder pain: a qualitative study. *Australian Journal of Physiotherapy*.

[29] Carter T, Hall H, McIntosh G, Murphy J, MacDougall J, Boyle C (2012). Intertester reliability of a classification system for shoulder pain. *Physiotherapy*.

[30] De Winter AF, Jans MP, Scholten RJPM, Devillé W, Van Schaardenburg D, Bouter LM (1999). Diagnostic classification of shoulder disorders: interobserver agreement and determinants of disagreement. *Annals of the Rheumatic Diseases*.

[31] Pellecchia GL, Paolino J, Connell J (1996). Intertester reliability of the Cyriax evaluation in assessing patients with shoulder pain. *Journal of Orthopaedic and Sports Physical Therapy*.

[32] Liesdek C, Van Der Windt DAWM, Koes BW, Bouter LM (1997). Soft-tissue disorders of the shoulder. A study of inter-observer agreement between general practitioners and physiotherapists and an overview of physiotherapeutic treatment. *Physiotherapy*.

[33] Wilson L, Hall H, McIntosh G, Melles T (1999). Intertester reliability of a low back pain classification system. *Spine*.

[34] Mathew B, Norris D, Hendry D, Waddell G (1988). Artificial intelligence in the diagnosis of low-back pain and sciatica. *Spine*.

[35] Ekeland AG, Bowes A, Flottorp S (2010). Effectiveness of telemedicine: a systematic review of reviews. *International Journal of Medical Informatics*.

[36] Huis in’t Veld MHA, van Dijk H, Hermens HJ, Vollenbroek-Hutten MMR (2006). A systematic review of the methodology of telemedicine evaluation in patients with postural and movement disorders. *Journal of Telemedicine and Telecare*.

[37] Åkesson KM, Saveman BI, Nilsson G (2007). Health care consumers’ experiences of information communication technology—a summary of literature. *International Journal of Medical Informatics*.

